# Whole exome sequencing reveals a biallelic frameshift mutation in *GRXCR2* in hearing impairment in Cameroon

**DOI:** 10.1002/mgg3.1609

**Published:** 2021-02-02

**Authors:** Ambroise Wonkam, Kamogelo Lebeko, Shaheen Mowla, Jean Jacques Noubiap, Mike Chong, Guillaume Pare

**Affiliations:** ^1^ Department of Medicine Division of Human Genetics Faculty of Health Sciences University of Cape Town Cape Town South Africa; ^2^ Institute of Infectious Diseases and Molecular Medicine Faculty of Health Sciences University of Cape Town Cape Town South Africa; ^3^ Department of Pathology Division of Haematology Faculty of Health Sciences University of Cape Town Cape Town South Africa; ^4^ Centre for Heart Rhythm Disorders South Australian Health and Medical Research Institute (SAHMRI University of Adelaide and Royal Adelaide Hospital Adelaide Australia; ^5^ Population Health Research Institute Hamilton Health Sciences and McMaster University Hamilton ON Canada

**Keywords:** Africa, Cameroon, *GRXCR2*, hearing impairment, whole exome sequencing

## Abstract

**Background:**

Hearing impairment (HI) genes are poorly studied in African populations.

**Methods:**

We used whole exome sequencing (WES) to investigate pathogenic and likely pathogenic (PLP) variants in 10 individuals with HI, from four multiplex families from Cameroon, two of which were previously unresolved with a targeted gene enrichment (TGE) panel of 116 genes. In silico protein modelling, western blotting and live imaging of transfected HEK293 cells were performed to study protein structure and functions.

**Results:**

All PLP variants previously identified with TGE were replicated. In one previously unresolved family, we found a homozygous frameshift PLP variant in *GRXCR2* (OMIM: 615762), NM_001080516.1(*GRXCR2*):c.251delC p.(Ile85SerfsTer33), in two affected siblings; and additionally, in 1/80 unrelated individuals affected with non‐syndromic hearing impairment (NSHI). The *GRXCR2*‐c.251delC variant introduced a premature stop codon, leading to truncation and loss of a zinc‐finger domain. Fluorescence confocal microscopy tracked the wild‐type GRXCR2 protein to the cellular membrane, unlike the mutated GRXCR2 protein.

**Conclusion:**

This study confirms *GRXCR2* as a HI‐associated gene. *GRXCR2* should be included to the currently available TGE panels for HI diagnosis.

## Graphical abstract

We used whole exome sequencing (WES) to investigate the pathogenic and likely pathogenic (PLP) variants in 10 individuals with HI, from four multiplex families from Cameroon, two of which were previously unresolved with a targeted gene enrichment (TGE) panel of 116 genes. All PLP variants previously identified with TGE were replicated. In one previously unresolved family, we found a homozygous frameshift PLP variant in *GRXCR2* (OMIM: 615762), *GRXCR2*‐c.251delC (p.Arg84 frameshift), in two affected siblings; and additionally, in 1/80 unrelated individuals affected with non‐syndromic hearing impairment (NSHI). The *GRXCR2*‐c.251delC variant introduced a premature stop codon, leading to truncation and loss of a zinc‐finger domain. Fluorescence confocal microscopy tracked the wild‐type GRXCR2 protein to the cellular membrane, unlike the mutated GRXCR2 protein. This study confirms*GRXCR2* as a HI‐associated gene.

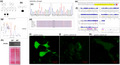

## 1 INTRODUCTION

Hearing impairment (HI) is the most common sensory disorder, affecting nearly 500 million people worldwide (WHO Media Centre, 2018). The highest incidence rate is found in sub‐Saharan Africa (SSA): up to 6 per 1000 compared to about 1 per 1000 in Europeans or Nord Americans (Olusanya et al., 2014). About 50% of congenital HI cases in high‐income countries are due to genetic causes (Schrijver, 2004). Variants in more than 150 genes have been associated with congenital HI (The Molecular Otolaryngology and Renal Research Laboratories, The University of Iowa, 2016), with common mutations in *GJB2* and *GJB6* associated with up to 50% non‐syndromic HI (NSHI) in Europeans and Asians (Chan & Chang, 2014). But mutations in *GJB2* and *GJB6* and other connexins genes have rarely been found in Africans with NSHI (Adadey et al., 2020; Bosch, Lebeko, et al., 2014; Bosch, Noubiap, et al., 2014; Gasmelseed et al., 2004; Javidnia et al., 2014; Kabahuma et al., 2011; Tingang Wonkam et al., 2019; Wonkam et al., 2015). An exception is the founder mutation NP_003995.2:p.(Arg143Trp) in the *GJB2* gene that accounts for 26% of familial and 8% of sporadic cases of NSHI in Ghana (Adadey et al., 2019; Hamelmann et al., 2001). In addition, targeted gene enrichment (TGE) panels have consistently displayed a lower pick up rate for HI in individuals of African ancestry (Rudman et al., 2017; Sloan‐Heggen et al., 2016; Yan et al., 2016). Moreover, the prevalence of autosomal recessive non‐syndromic hearing impairment (ARNSHI) due to pathogenic and likely pathogenic (PLP) variants, selected from the ClinVar and Deafness Variation Databases, and gnomAD database, was estimated at 5.2 per 100,000 individuals for Africans/African Americans, compared to the highest prevalence of 96.9 per 100,000 individuals for Ashkenazi Jews (Chakchouk et al., 2019). This knowledge deficit in genetic cause of HI in populations of African ancestry is likely to hinder our current understanding of hearing pathophysiology, refinement of gene‐disease pairs and clinical validity curation (DiStefano et al., 2019).

We previously used a TGE panel of 116 genes (OtoSCOPE®) to successfully resolve the genetic causes in 7/9 selected multiplex families, segregating ARNSHI from Cameroon (Lebeko et al., 2016). In the present study, we use whole exome sequencing (WES) to investigate the causative variants in the two unresolved families, while exploring its sensitivity in detecting variants found in two other families through TGE. We further used in silico analyses to predict disruptions in protein folding and partner interactions, and in vivo cellular assays to explore and visualize protein localization.

## 2 METHODS

### 2.1 Ethical approval

The study was performed in accordance with the Declaration of Helsinki. This study was granted ethics approval by the Cameroon National Ethics Committee (ethics approval N°123/CNE/SE/2010 and N°033/CNE/DNM/07) as well as the University of Cape Town Human Research Ethics Committee (ethics approval HREC REF: 455/2014 and HREC REF: 132/2010). Written informed consent was obtained from patients 18 years or older or from parents/guardians for minors with verbal assent from the children.

### 2.2 Patient participants

Four multiplex Cameroonian families of 10 members affected with ARNSHI were selected for WES, all previously investigated with TGE performed at the Molecular Otolaryngology and Renal Research Laboratories, Carver College of Medicine, University of Iowa, Iowa City, USA, as previously reported (Lebeko et al., 2016). In order to explore the sensitivity of WES, these families included two in which no causative variants were found using a TGE panel of 116 genes (OtoSCOPE^®^), and two other families in which the causative variants were known.

Unrelated participants with sporadic and familial NSHI (*n* = 80) were investigated for allele frequency (AF) of potential PLP variants found with WES. These participants were recruited in Cameroon (*n* = 57) and South Africa (*n* = 23). DNA samples were extracted from peripheral blood samples, and all participants did not have PLP variants in *GJB2* and *GJB6*, following direct Sanger sequencing performed at the Division of Human Genetics, Faculty of Health Sciences, University of Cape Town, South Africa, as previously reported (Bosch, Noubiap, et al., 2014; Wonkam et al., 2013).

### 2.3 Control participants

Ethnically matched control participants (*n* = 100) were recruited in Cameroon from an apparently healthy group of blood donors, without a personal or family history of HI, at Central Hospital of Yaounde, Cameroon.

### 2.4 Whole exome sequencing

A total of 10 individuals with ARNSHI from four unrelated multiplex families underwent WES. DNA was extracted from peripheral blood samples as previously reported (Bosch, Noubiap, et al., 2014). Using the SureSelect Human All Exon 50 Mb kit (Agilent Technologies, Inc.), which covers the exonic sequences of ≈24 000 genes corresponding to 50 Mb of genomic DNA, library preparation and sequencing on the Illumina HiSeq 2000 were performed at the Institute of Human Genetics, Helmholtz Zentrum München, Germany. The Burrows–Wheeler Alignment tool (version 0.7.5) was used to align the reads to the human genome assembly hg19 (GRCh37). Variant calling was performed with SAMtools (v0.1.19) and PINDEL (v0.2.4t). Variant quality was determined using the SAMtools varFilter script: default parameters were applied, except for the minimum *P* value for base quality bias (−2), which was set to 1e−400. Variant annotation was performed by applying custom Perl scripts, including information about known transcripts (UCSC genes and RefSeq genes), known variants (dbSNP v135), type of variant, and amino acid change (where applicable). Variants were filtered and excluded if there was no AF information in DBSNP137, 1000KG201204, ESP6500AA and ESP6500EA databases. PLP variants were evaluated and defined according to consensus criteria of the American College of Medical Genetics and Genomics and the Association for Molecular Pathology (Richards et al., 2015).

### 2.5 Genotyping of targeted variants

To investigate any other potentially causative and secondary variants in *GRXCR2*, which were found through WES to have a PLP variant in one family, the entire coding region of *GRXCR2* was screened using direct Sanger sequencing in 80 patients with NSHI, and 100 Cameroonian controls, at the Division of Human Genetics, Faculty of Health Sciences, University of Cape Town, South Africa. Primers were designed to amplify all three exons of *GRXCR2*. PCR amplification was confirmed by gel electrophoresis and products were cleaned up using FastAp and Endonuclease I. BigDye^®^ Terminator v3.1 Cycle Sequencing mix was used for the sequencing reaction according to the manufacturer's guidelines (Thermo Fisher Scientific). Sequencing products were resolved on 3130xl Genetic Analyser ABI Prism using capillary electrophoresis and resultant electropherograms were analysed using DNAStar software (Applied Biosystems).

### 2.6 In silico pathogenicity prediction of genomic variants

The *GRXCR2* cDNA sequence (ENST00000377976.2) was extracted from the Ensemble genome browser and manually edited by deleting the cytosine at position 251 of the cDNA sequence. The altered sequence was then interrogated using two online programs, namely ExPASy (https://web.expasy.org/translate/) and EMBOSS Transeq (http://www.ebi.ac.uk/Tools/st/emboss_transeq/). This was done to predict the effect of the identified novel frameshift deletion on the translated protein sequence. To infer the importance of this variant across species, a multiple sequence alignment of higher primates was extracted from Ensemble (http://ensembl.org).

### 2.7 Protein prediction and modelling

The PSIPRED protein sequence analysis workbench online platform (PSIPRED v3.3 tool: http://bioinf.cs.ucl.ac.uk/psipred/) (Buchan et al., 2013) was used to predict protein secondary structures and intrinsically disordered regions (IDRs). Protein–protein interactions (PPIs) and the potential network of *GRXCR2* were investigated using STRING (https://string‐db.org/).

### 2.8 In vitro functional analysis of the *GRXCR2* variant

#### 2.8.1 *GRXCR2* plasmid constructs

To create the NM_001080516.1(*GRXCR2*):c.251delC variant, the pCMV6‐*GRXCR2*‐WT (wild type) plasmid (Myc‐DDK‐tagged) (OriGene—RC213752) was modified as follows. Site‐directed mutagenesis (SDM) was performed using an adapted protocol supplied by the Stratagene QuikChange system, and the primer pairs used were 5′‐ACTGCTCAGAGATCAGTGTGTTTAGAGAGGG‐3′ and 5′‐CCCTCTCTAAACACACTGATCTCTGAGCAGT‐3′. Successful introduction of the mutation was confirmed by automated BigDye Terminator Sanger sequencing (Applied Biosystems) as described above. The pCMV‐GRXCR2‐WT and pCMV‐*GRXCR2*‐MT (mutant) vectors were generated by amplifying the open reading frames (ORFs) from the pCMV6‐GRXCR2 plasmid by PCR using a forward primer harbouring a HindIII site, (5′‐GCCGCCAAGCTTCCATGGAGG‐3′) and a reverse primer harbouring a BamHI site (5′‐CCGCGTGGATCCTTGATTGCA‐3′), via sub‐cloning into the pGEM^®^‐T Easy vector (Promega). Cloning was confirmed through direct Sanger sequencing.

#### 2.8.2 Expression and detection of FLAG‐tagged GRXCR2 in HEK293 cells

##### Cell line and transfection

The HEK293 Human Embryonic Kidney cell line was maintained in Dulbecco's modified Eagle medium (DMEM) supplemented with 10% (v/v) foetal bovine serum (FBS), 200 units/ml penicillin and 100 μg/ml streptomycin. Transient transfections were performed using X‐tremeGENE™ HP DNA Transfection Reagent according to the manufacturer's instructions (Roche). Cells were plated at 3.5 × 10^4^ cells/well in 35 mm diameter dishes 16 h before transfection. Cells were transfected with 1 µg of pCMV6‐*GRXCR2*‐WT or pCMV6‐*GRXCR2*‐MT and cultured for 30 h before total protein harvest. For microscopy visualization, cells were plated as before, and transfected with 2 µg pCMV‐*GRXCR2*WT or pCMV‐*GRXCR2*MT or pEGFP‐C1 and incubated for 48 h before being prepared for viewing.

### 2.9 Western blot analysis

Whole cell extracts were prepared using boiling blue buffer (125 mm Tris‐HCL‐pH 6.8; 4% SDS; 10% β‐mercaptoethanol; 20% glycerol). Proteins were resolved by SDS/PAGE (12%) and transferred to Hybond ECL membranes (Amersham Biosciences). Ponceau S stain was used to verify transfer. The membrane was probed with the mouse monoclonal anti‐FLAG M2 primary antibody (Sigma Aldrich F1804; 1:1000), detected using peroxidase‐conjugated anti‐mouse secondary antibody and visualized by enhanced chemiluminescence (ECL) (Bio‐Rad Laboratories).

### 2.10 Visualization using confocal microscopy

Live viewing was performed 48 hours following transfection, using a Zeiss LSM8800 with Airyscan confocal microscope (Zeiss). The detector of the confocal was a photo multiplier tube (PMT) and allowed detection of the green fluorescence signal through the Argon laser at 488 nm. Images were visualized and processed using the ZEN Black Software (latest version) provided by Zeiss (Zeiss).

## 3 RESULTS

### 3.1 Description of families selected for WES

The pedigrees of multiplex families selected for WES are shown in Figure S1. All affected members presented with autosomal recessive bilateral sensorineural non‐syndromic HI, with variable severity.

### 3.2 PLP found with TGE panel replicated with WES

WES confirmed PLP variants previously found in two families: homozygous NM_194248.3 (*OTOF*):c.766‐2A>G, and compound heterozygous NM_000441.2(*SLC26A4*):c.[1678G>A];[2007C>A] respectively (Table 1). In one family, novel variants were identified through WES in *PAX3* and *MYO7A*, but were excluded on account of their possible syndromic manifestation as well as their suspected dominant pattern of inheritance, both of which did not fit the ARNSHI phenotype observed in the family. No causative variants were identified for one family through TGE and WES.

**TABLE 1:** Pathogenic and likely pathogenic (PLP) variants found with targeted gene enrichment (TGE) panel (OtoSCOPE®), and whole exome sequencing (WES)

Multiplex families segregating hearing Impairment		OtoSCOPE®			WES findings		
		Gene	Nucleotide change	Protein change	Gene	Nucleotide change	Protein change
4	Causative variants	*OTOF* (OMIM: 603681)	c.766‐2A>G g.68624A>G	Intronic	*OTOF* (OMIM: 603681)	c.766‐2A>G g.68624A>G	intronic
	Secondary findings	*CDH23* (OMIM: 605516)	c.3361A>T	p.Ile1121Phe			
		*GSDME* (OMIM: 608798)	c.658G>A	p.Gly220Ser			
		*TMPRSS3* (OMIM: 605511)	c.715T>C	p.Try239His			
		*USH2A* (OMIM: 608400)	c.14804G>A	p.Arg4935Gln			
		*USH2A* (OMIM: 608400)	c.12883A>G	p.Ile4295Val			
		*USH2A* (OMIM:608400)	c.4796G>A	p.Gly1599Asp			
6	Causative variants	*SLC26A4* (OMIM: 605646)	c.1678G>A	p.Asp560Asn	*SLC26A4* (OMIM: 605646)	c.1678G>A	p.Asp560Asn
			c.2007C>A	p.Asp669Glu		c.2007C>A	p.Asp669Glu
		*STRC* (OMIM: 606440)	Complex Copy Number Variation	‐			
	Secondary findings	*CDH23* (OMIM: 605516)	c.9758A>C	p.Asp3253Ala	*PAX3* (OMIM: 606597)	c.1320C>T	p.Asp440=
		*GJB3* (OMIM: 603324)	c.300G>C	p.Glu100Asp	*MYO7A* (OMIM: 276903)	c.4878_4879insT	p.Ala1628CysfsTer11
		*MYO7A* (OMIM: 276903)	c.6002C>T	p.Thr2001Met	*MYO7A* (OMIM: 276903)	c.6002C>T	p.Thr2001Met
		*TRIOBP* (OMIM: 609761)	c.3068C>T	p.Ala1023Val			
		*WFS1* (OMIM: 606201)	c.854G>A	p.Arg285His			
8	Causative variants	*‐*			*GRXCR2* (OMIM: 615762)	c.251delC	p.Ile85SerfsTer33
	Secondary findings	*CDH23* (OMIM: 605516)	c.6329C>T	p.Ala2110Val	‐		
		*CDH23* (OMIM: 605516)	c.6596T>A	p.Ile2199Asn			
		*ADGRV1* (OMIM: 602851)	c.463A>G	p.Ile155Val			
		*MYO7A* (OMIM: 276903)	c.5065G>A	p.Asp1689Asn			
		*TPRN* (OMIM: 613354)	c.1259C>T	p.Pro420Leu			
		*TRIOBP* (OMIM: 609761)	c.202A>G	p.Thr68Ala			
		*USH2A* (OMIM: 608400)	c.13409C>T	p.Pro4470Leu			
9	Causative variants	—		—			
	Secondary findings	*ADGRV1* (OMIM: 602851)	c.6383G>A	p.Arg2128Gln	‐		
		*LRTOMT* (OMIM: 612414)	c.*1867T>A	Intronic			
		*MYH14* (OMIM: 608568)	c.262C>T	p.Arg88Trp			
		*MYO15A* (OMIM: 602666)	c.3359G>A	p.Arg1120His			
		*MYO7A* (OMIM: 276903)	c.*546C>T	Intronic			
		*USH1C* (OMIM: 605242)	c.1069C>T	p.Arg357Trp			
		*USH2A* (OMIM: 608400)	c.8575C>T	p.Arg2859Cys			
		*WFS1* (OMIM: 606201)	c.482G>A	p.Arg161Gln			

RefSeq: *ADGRV1*, NM_032119.4; *CDH23*, NM_022124.6; *GJB3*, NM_001005752.2; *GRXCR2*, NM_001080516.1; *GSDME*, NM_001127453.2; *LRTOMT*, NM_001145308.5; *MYH14*, NM_001145809.2; *MYO7A*, NM_000260.4; *MYO15A*, NM_016239.4; *OTOF*, NG_009937.1; *PAX3*, NM_181459.4; *SLC26A4*, NM_000441.2; *STRC*, NM_153700.2; *TMPRSS3*, NM_001256317.2; *TPRN*, NM_001128228.3; *TRIOBP*, NM_001039141.3; *USH1C*, NM_153676.4; *USH2A*, NM_206933.4; *WFS1*, NM_006005.

### 3.3 Homozygous *GRXCR2*‐c.251delC (p.Arg84 fs) in one family, and an unrelated case with NSHI

**FIGURE 1:**
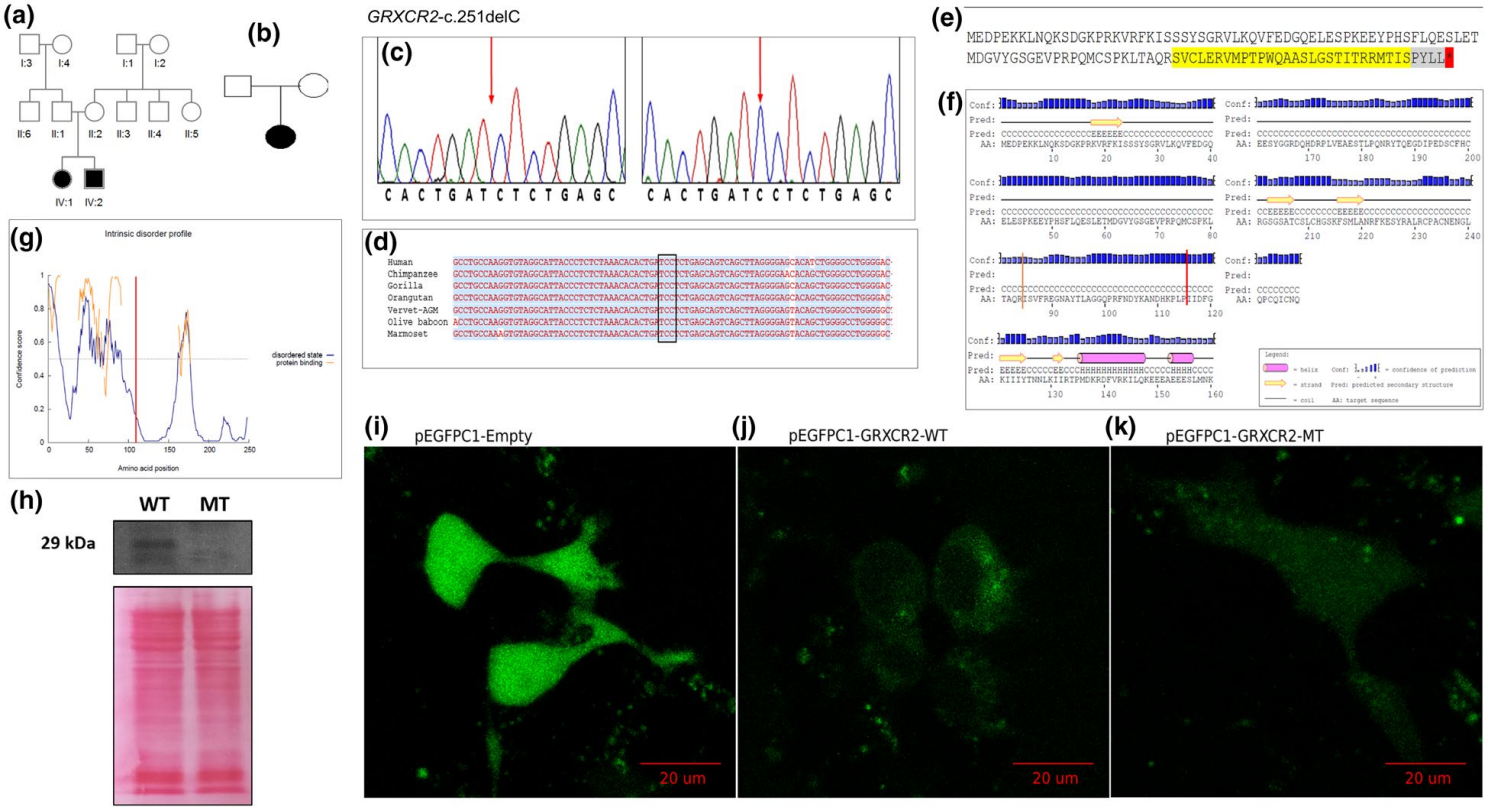
*GRXCR2*‐c.251delC (p.Arg84fs) is associated with HI in Cameroonian patients. Panel a: Pedigree of the multiplex family compatible with autosomal recessive hearing impairment due to a biallelic *GRXCR2*‐c.251delC p.(Ile85SerfsTer33) mutation found on whole exome sequencing of the two affected siblings. Panel b: Pedigree of the sporadic non‐syndromic hearing impairment case with a biallelic *GRXCR2*‐c.251delC p.(Ile85SerfsTer33) mutation, from a singleplex Cameroonian family, found with direct Sanger sequencing. Panel c: Partial chromatograms of exon one: Sanger sequencing results validating the presence of the *GRXCR2*‐c.251delC variant in patients, and the retention of the cytosine in a control sample. Panel d: Evolutionary conservation of portion of *GRXCR2* in higher primate species with affected codon showing conservation: The sequence of the codon and surrounding codons have been maintained across these higher primates which can infer the biological and functional importance of the base. Panel e: Protein translation sequence of GRXCR2 in the presence of the c.251delC variant. Yellow highlighted residues indicate residues which are altered in exon 1. Grey highlighted bases are altered in exon 2 and the *denotes the premature stop codon. Panel f: Secondary structures of GRXCR2 by PSIPRED. Orange vertical line denotes the site of the base deletion and alteration of protein sequence. The red vertical line denotes the position of the premature stop codon. Panel g: DISOPRED3 Intrinsic Disorder profile of GRXCR2 with protein binding regions. Red vertical line denotes truncation of protein. Given the presence of protein binding sequences or regions within GRXCR2, potential predicted protein–protein interactions (PPIs) were investigated using STRING database, but no data were available for GRXCR2. Panel h: The Ponceau stain showing successful transfer of protein from gel to nitrocellulose and equal loading of wild‐type and mutant proteins. Lane wild‐type (WT) is loaded with total protein lysate from cells transfected with wild‐type ORF. Lane Mutant (MT) is the protein lysate from cells transfected with the mutated GRXCR2 ORF. The GRXCR2‐c.251delC mutation prevents detection of DDK/FLAG: HEK293 cells were transfected with the pCMV‐GRXCR2‐WT or GRXCR2‐MT expression constructs. Western analysis of whole cell protein lysates from the transfected cells indicated GRXCR2‐WT steady state levels were detectable using an antibody against DDK/FLAG, while this was not the case for the GRXCR2‐MT protein. Panel i: Representative cells showing expression of GFP in HEK293 cells: HEK293 cells showing different intensities of uniform GFP expression when transfected with the GFP‐empty vector. Panel j: Representative cell showing expression of GFP‐GRXRC2 wild type (WT) in HEK293 cells: panels show distinct darker regions owing to shuffling of recombinant protein out of the nucleus. Panel k: GFP with mutant (MT) GRXCR2 shows uniform expression with decreased intensity

In one multiplex family (Figure 1a), WES identified a homozygous *GRXCR2*‐c.251delC p.(Ile85SerfsTer33) at position 5:145252279 in the GRCh37 human reference genome build. The variant was identified in one other unrelated sporadic Cameroonian proband (Figure 1b) with non‐symmetrical NSHI (profound in the left ear and severe in the right ear). This variant was not found in 100 Cameroonian controls. *GRXCR2 *has three exons, all of which are coding, and translates into a 248‐residue‐long protein of a size of about 28 kDa. The *GRXCR2*‐c.251delC variant is in exon 1 and was confirmed by Sanger sequencing (Figure 1c). There was an evolutionary conservation of this base across eight primate species (Figure 1d).

### 3.4 Screening of *GRXCR2* for additional variants in HI patients through Sanger sequencing

Identification of the identified novel variant in a second unrelated case (Figure 1b) prompted the sequencing of the other two exons of *GRXCR2* in 80 patients with NSHI from Cameroon and South Africa. The sociodemographic and phenotypic description of these patient are summarized in Tables S1 and S2. Sixty‐five percent (65%) of the patients were males (*n* = 52) with 70% of patients reported as having a prelingual onset of hearing loss. Up to 47.5% (*n* = 38) were reported as having a family history of hearing loss, with most of this group being from Cameroon. All the Cameroonian patients had sensorineural NSHI of various degrees, symmetrical in most cases (*n* = 40). No other PLP variants were found in *GRXCR2*, but two polymorphisms were identified in patients and controls (Table S3).

### 3.5 GRXCR2 protein analysis

Protein sequence prediction by ExPASy and EMBOSS Trans revealed that the *GRXCR2*‐c.251delC p.(Ile85SerfsTer33) altered the amino acid sequences after p.Arg84 as well as the introduction of a premature stop codon at position 117 of the amino acid sequence (Figure 1e). In silico analysis of the secondary structure of the GRXCR2 protein depicted the predicted domains and their positions along the protein sequence, showing the mutant GRXCR2 protein to be a relatively small protein with mostly random coiling with few helices and beta sheets (Figure 1f). The mutant sequence could not be interrogated in PSIPRED due to its small size. The predicted stability was explored (Figure 1g) according to identified IDRs, which are regions that can alter their state from structured to unstructured as a protein prepares to or carries out its functions, particularly in binding with other proteins. GRXCR2 was found to have three distinct protein binding regions corresponding with high confidence of IDRs. Truncation of the protein leads to the loss of the C‐terminus IDR which includes the cysteine‐rich region, which is predicted to fold into a zinc‐finger structure known to act in PPIs (Figure 1g).

### 3.6 Functional Studies

#### 3.6.1 Confirmation of premature stop codon using western blotting

To confirm the predicted truncation of the mutant protein, western blotting was used. The DDK‐tag located on the C‐terminal end of the protein could be detected in the wild‐type (WT) protein, showing a predicted size of 24 kDa, but the same was not present in the mutant (MT) protein, indicating premature truncation (Figure 1h).

#### 3.6.2 Live imaging shows disruption of protein transport and localization of WT compared to MT GRXCR2 proteins

The WT and MT proteins could be visualized via N‐terminal GFP tags, using confocal microscopy in HEK293 cells. As expected, cells transfected with the empty vector showed strong and uniform pattern of expression throughout the HEK293 cells (Figure 1i). On the other hand, the WT‐GTP‐tagged GRXCR2 protein was preferentially localized outside of the nucleus and predominantly in the cytoplasm, with some punctate staining close to the periphery of the cell membranes (Figure 1j). This suggests that the WT protein is expressed in the cytoplasm, where it is confined, and potentially shuttled to the periphery close to the membrane. Since these cells were visualized live to preserve the GFP, a DNA‐specific stain could not be used to locate the nuclei. Interestingly, and in contrast to the GFP‐GRXCR2‐WT protein, the GRXCR2‐MT protein lacked any particular localization within the cells and showed a distribution similar to that observed for the empty vector, although at a seemingly lower intensity (Figure 1k).

## 4 DISCUSSION

To date, only a few papers have explored the use of TGE in HI in SSA populations (Bademci et al., 2016; Lebeko et al., 2016; Rudman et al., 2017), often with lower pick‐up rate compared to European and Asian populations. The present study is therefore a rare attempt to use WES to investigate HI in multiplex families from SSA (Cameroon), showing the efficiency of this approach in being specific enough to identity variants in known genes, but equally sensitive enough to identify variants in novel HI genes. The present report from an understudied African population is important for improving the disease‐gene pair curation, globally. Indeed, PLP variants in *GRXCR2* were reported only twice: the *GRXCR2*‐c.714dupT mutation was identified in a Pakistani family segregating ARNSHI (Imtiaz et al., 2014), and *GRXCR2*‐c.65A>G was recently reported in Chinese proband with HI (Wu et al., 2020). Therefore, this study provides additional evidence to confirm *GRXCR2* as an ARNSHI gene in humans. Interestingly, null mutations in *GRXCR2* in mice result in early onset progressive hearing loss without vestibular dysfunction (Avenarius et al., 2018). The *GRXCR2*‐c.251delC mutation reported in the present study is absent from gnomAD v3.0, which includes >20,000 African exomes or genomes. Generally, the occurrence of frameshift mutations in this gene is very rare (cumulative minor allele frequency (CMAF) in gnomAD overall = 4.89 × 10^−5^; CMAF in African = 7.14 × 10^−5^), and there are no homozygote carriers for any frameshift mutation (though we cannot exclude the possibility of compound heterozygote carriers). Based on gnomAD, the frequency of biallelic carriers for *GRXCR2* frameshift mutations is exceptionally rare at 2.19 × 10^−9^. The NHIBI Exome Sequencing Project reports a single heterozygous carrier of the *GRXCR2*‐c.251delC variant reported in this paper, in an African American individual (http://evs.gs.washington.edu/EVS/), suggesting that investigating sub‐Saharan Africans will have implications for the African diaspora. Transcript of the inner hear indicates that 100 s of HI genes are still to be discovered (Hertzano & Elkon, 2012).

In the present study, both in silico and in vitro investigations (Figure 1e–k) support the contribution of the *GRXCR2*‐c.251delC variant in HI in the patients investigated. The *GRXCR2* gene is expressed in the inner ear during development (Schraders et al., 2010). The GRXCR2 protein belongs to the glutaredoxin domain‐containing family. It is a paralog of *GRXCR1* and both have been shown to be required for stereocilia bundle development, organization and maintenance (Avenarius et al., 2018). Pirouette mouse models showed variants in *GRXCR1* to result in hearing loss with vestibular dysfunction (Odeh et al., 2010). A similar syndromic phenotype was observed in humans who carried pathogenic variants in the *GRXCR1* gene (Schraders et al., 2010). The differences observed in the phenotype of variants of *GRXCR1* and *GRXCR2* suggest that the proteins they encode might have slightly distinct roles in the stereocilia maturation pathway (Avenarius et al., 2018; Liu et al., 2018). It is most likely that PPIs mediated by the domain missing in the protein encoded by the *GRXCR2*‐c.251delC variant are essential for the protein's functionality, with *GRXCR1*, particularly in the formation of homodimers or other protein complexes (Avenarius et al., 2018). Functional interaction between the *GRXCR2*‐ and *GRXCR1*‐*encoded proteins* could be similar to that seen in the Connexin family between *GJB2*‐ and *GJB6*‐encoded proteins, where their heterodimerization is needed for functionality (Xu & Nicholson, 2013), and deserves additional exploration.

We could not identify PLP variants in one multiplex family. This could be due to a complex structural change which was not detected by WES, or variants in other parts of the genome. The closest approach to whole‐genome sequencing (WGS) was whole‐genome SNP mapping, which was able to resolve 100% of 30 families with HI from Pakistan (Shafique et al., 2014). However, this might not be as effective in a population of African ancestry, which was poorly represented in the data that informed the development of panels used (Lek et al., 2016).

This study has a few limitations: firstly, the *GRXCR2*‐c.251delC variant was not investigated in parents or other family members due to lack of available DNA. Secondly, in our functional studies, the use of GRXCR2‐specific antibodies could have increased accuracy of both localization and protein expression levels, and future study should demonstrate that the mutation caused a truncated protein via inability to detect the tag on the C‐terminus of the protein. Moreover, the identification of the same homozygous mutation in patients from non‐consanguineous unrelated families suggests the presence of a founder effect or a mutational hotspot that could be investigated in future. Despite these limitations, the study has provided sufficient data to support the implication of *GRXCR2* in HI in Cameroonian patients investigated.

## 5 CONCLUSIONS

This study has provided additional evidence to confirm *GRXCR2* as a HI‐associated gene. *GRXCR2* should be included to the currently available TGE panels for HI diagnosis. Additionally, the study showed that WES is as sensitive as TGE in detecting variants in known HI genes, and emphasizes the urgent need to use WES to enhance HI genes and discovery of variants in populations of African ancestry.

## CONFLICT OF INTEREST

The authors report no conflict of interest. The authors alone are responsible for the content and writing of this article.

## AUTHOR CONTRIBUTIONS

AW and SM contributed to conception and design of the study; KL, JJNN and AW collected data; KL, SM, JJNN AW, MC and GP performed molecular analysis, MC and GP performed the WES interpretation of data; AW, KL and SM wrote the first draft of the manuscript. All authors contributed to manuscript revision, read and approved the submitted version.

## Supporting information

Fig S1‐Table S1‐S3

## Data Availability

The datasets used and/or analysed during the current study are available from the corresponding author on reasonable request.
