# Supplementary material for: Whole exome sequencing reveals a biallelic frameshift mutation in GRXCR2 in hearing impairment in Cameroon
Source: Mol Genet Genomic Med. 2021 Feb 2;9(3):e1609. doi: 10.1002/mgg3.1609 (PMC8104159; doi:10.1002/mgg3.1609)
Supplement: Supplementary file 1 — Fig S1‐Table S1‐S3 [file MGG3-9-e1609-s001.pdf]

Supplementary Figure

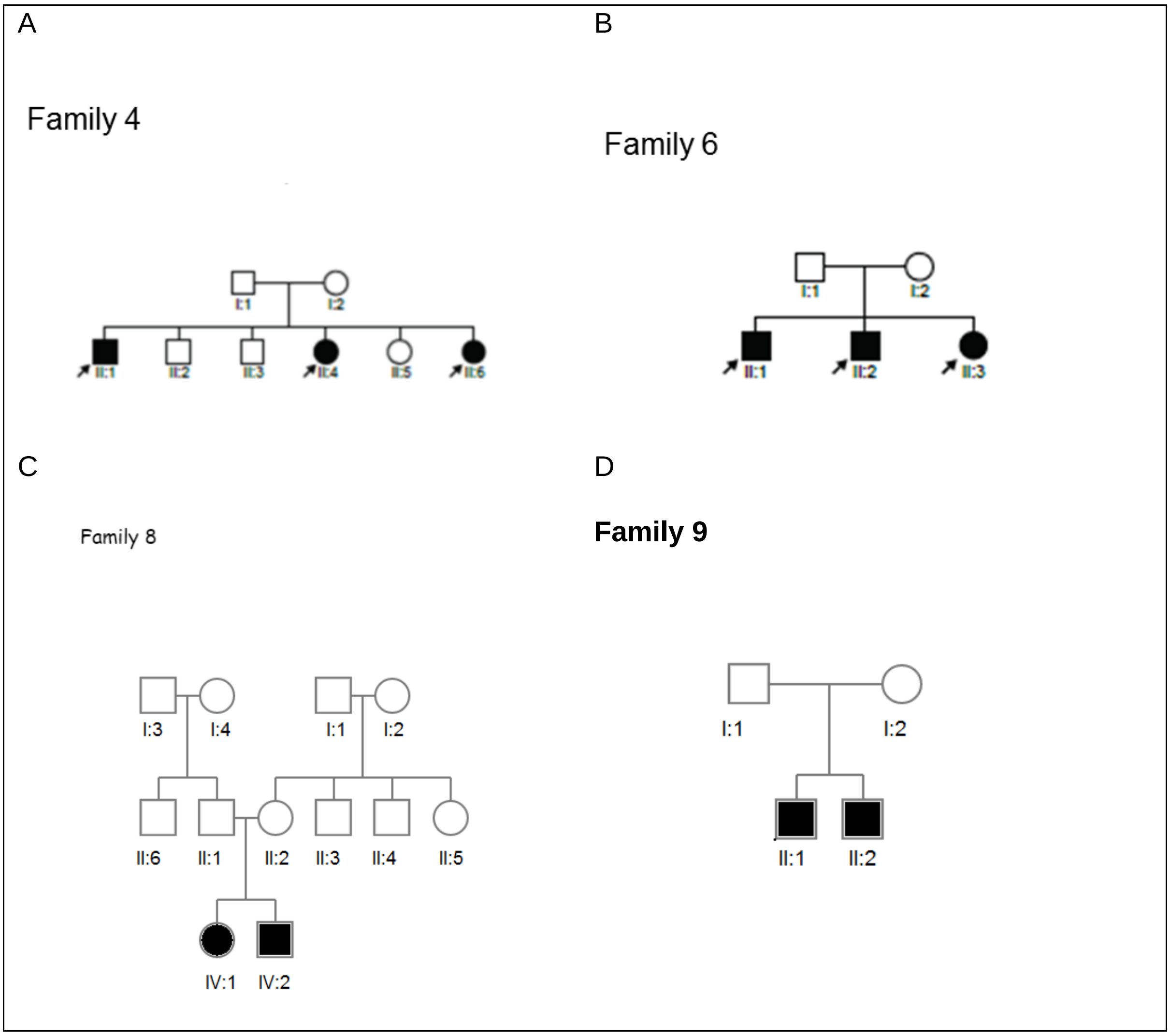

Figure S1: Pedigrees of multiplex families selected for WES, following TGE

All families show at least two affected children born to unaffected parents, compatible with an autosomal recessive mode of inheritance. We use whole exome sequencing (WES) to investigate the causative variants in the two families (families 8 and 9) in which causative mutations were not found through targeted gene enrichment (TGE) panel of 116 genes (OtoSCOPE®), while exploring WES sensitivity in detecting causative variants previously found in two other families through TGE (family 4 and 6).

Supplementary Tables

Table S1: Summary of socio-demographic data of patients

		South African (n= 23)	Cameroon (n=57)	Total (n=80)
**Sex**	**Male**	19	33	52
	**Female**	4	24	28
**Age of Onset**	**Prelingual**	3	53	56
	**Perilingual**	6	-	6
	**Postlingual**	3	3	6
	**Undetermined**	11	1	12
**Transmission**	**Familial (autosomal recessive)**	5	33	38
	**Sporadic**	13	21	24
	**Undetermined**	5	3	8

Prelingual – Before the development of speech; Perilingual - Onset before speech development is completed; Postlingual – Onset occurred after the development of speech; Undetermined – No data is available in records.

Table S2: Degree of hearing loss of Cameroonian patients

Degree of hearing loss (dB)	Cases	%
**Severe 1 (71 – 80)**	2	2.5
**Severe 2 (81 – 90)**	6	7.5
**Profound 1 (91 – 100)**	20	25
**Profound 2 (101 – 110)**	16	20
**Profound 3 (111 – 120)**	7	8.75
**Total HL (>120)**	1	1.25
**Not determined**	28	35
**Total**	80	100

Degree of hearing loss categories as provided by WHO. Patients are classified according the degree of hearing loss observed in the better hearing ear in cases of non-symmetrical hearing loss.

Table S3. Variants identified in exon 2 and exon 3 of GRXCR2 from sequencing of the entire patient cohort

Genomic position	Nucleotide change	Protein change	Pathogenicity	Cameroonian Alleles (homozygous)	South African Alleles (homozygous)
5:145866522	c.543A>C	p.Leu181Phe	Tolerated	10/114 (2)	5/46 (0)
5:145866555	c.510C>T	p.His170=	Benign	4/114 (0)	0/46

RefSeq: GRXCR2, NM_001080516.1
